# The Relation of Angiotensin-Converting Enzyme 2, Renin-Angiotensin-Aldosterone System Inhibitors, and Arterial Stiffness in Acute COVID-19 Emergency Department Patients—A Prospective Observational Study

**DOI:** 10.3390/jcm14072233

**Published:** 2025-03-25

**Authors:** Sebastian Schnaubelt, Anna Jakobljevich, Roman Brock, Julia Oppenauer, Andrea Kornfehl, Felix Eibensteiner, Christoph Veigl, Thomas Perkmann, Helmuth Haslacher, Robert Strassl, Roman Reindl-Schwaighofer, Oliver Schlager, Patrick Sulzgruber

**Affiliations:** 1 Department of Emergency Medicine, Medical University of Vienna, 1090 Vienna, Austria; 2 Emergency Medical Service Vienna, 1030 Vienna, Austria; 3 Division of Pulmonology, Department of Internal Medicine II, Medical University of Vienna, 1090 Vienna, Austria; anna.jakobljevich@meduniwien.ac.at; 4 Department Laboratory Medicine, Medical University of Vienna, 1090 Vienna, Austria; 5 Division of Clinical Virology, Department of Laboratory Medicine, Medical University of Vienna, 1090 Vienna, Austria; 6 Division of Nephrology, Department of Internal Medicine III, Medical University of Vienna, 1090 Vienna, Austria; 7 Division of Angiology, Department of Internal Medicine II, Medical University of Vienna, 1090 Vienna, Austria; 8 Division of Cardiology, Department of Internal Medicine II, Medical University of Vienna, 1090 Vienna, Austria

**Keywords:** COVID-19, RAAS inhibitors, ACE2, arterial stiffness, ankle-brachial index, pulse-wave velocity

## Abstract

**Background**: Severe acute respiratory syndrome coronavirus 2 (SARS-CoV-2) causing coronavirus disease 2019 (COVID-19) can damage the endothelium and increase arterial stiffness, potentially leading to adverse cardiovascular events. In parallel, systemic inflammation in COVID-19 also impacts endothelial function. Angiotensin-converting enzyme 2 (ACE2) promotes vasodilation and anti-inflammatory effects, but also facilitates SARS-CoV-2 entry into human cells. Thus, concerns have been raised about the use of RAAS inhibitors (RAASi) in COVID-19 patients due to potential ACE2 upregulation. However, the clinical significance of increased plasma ACE2 (sACE2) in RAASi-treated COVID-19 patients remains unclear. **Methods**: This prospective, single-centre study evaluated RAASi, sACE2, and vascular function in acutely ill patients with COVID-19 in comparison with acutely ill patients without COVID-19. Adult emergency department patients with confirmed or suspected COVID-19 were enrolled and underwent pulse wave velocity, ankle brachial index, and sACE2 measurements. **Results**: In the 152 included patients (50% female, median age 62 years, 68% COVID-19 positive), the sACE2 values were slightly higher in the COVID-19 (0.485 [0.364–1.329]) than in the non-COVID-19 subgroup (0.458 [0.356–1.138]; *p* = 0.70). No significant differences in sACE2 were observed between patients with and without RAASi, regardless of COVID-19 status. Pulse wave velocity values differed significantly between groups (*p* = 0.015). **Conclusions**: In emergency department patients, sACE2 was upregulated in COVID-19 patients, probably due to oxidative stress and inflammation. RAASi did not increase sACE2, but may have protective effects against inflammation. Elevated sACE2 appeared to have a beneficial effect on arterial stiffness in all patients. These findings support continued RAASi therapy in COVID-19 patients to protect against chronic inflammation and apoptosis.

## 1. Introduction

Since the onset of the coronavirus disease 2019 (COVID-19) pandemic in December 2019, caused by severe acute respiratory syndrome coronavirus 2 (SARS-CoV-2), the virus has led to several million deaths worldwide [1]. Angiotensin-converting enzyme 2 (ACE2) was identified as the receptor for SARS-CoV in 2003 and later for SARS-CoV-2 in 2020, facilitating the virus’s entry into human cells [2,3,4]. The receptor-binding domain (RBD) of SARS-CoV-2 exhibits an approximate 10- to 20-fold higher affinity to human ACE2 compared with SARS-CoV, contributing to the virus’s higher infectivity. ACE2 is highly expressed in alveolar epithelial cells, making the lungs a primary target for SARS-CoV-2. However, ACE2 receptors are distributed throughout various human organs, increasing the risk for diverse complications as the virus spreads beyond the respiratory system to affect the vascular and nervous systems [3,5,6,7,8,9,10]. SARS-CoV-2 can significantly impact the cardiovascular system, potentially leading to life-threatening conditions like myocardial injury, myocarditis, dysrhythmia, and venous thromboembolism. The virus can cause endothelial damage and increase arterial stiffness, which in turn can result in major adverse cardiovascular events (MACE) with a high risk of mortality [10,11,12].

Arterial thickening and stiffening, commonly associated with aging, can cause insufficient oxygen supply and tissue perfusion, increasing the risk for cardiovascular diseases like myocardial infarction or stroke. Systemic inflammation in COVID-19 may contribute to increased arterial stiffness due to endothelial damage: The vascular endothelium is harmed by systemic hyper-inflammation and cytokine release, induced by SARS-CoV-2. Due to acute systemic inflammation, nitric oxide (NO) bioavailability is reduced by the cytokine cascade, which henceforth increases arterial stiffness [9,13,14,15].

The renin−angiotensin−aldosterone system (RAAS) plays a critical role in vascular function regulation. Angiotensin II (Ang II), produced through the conversion of angiotensinogen by renin and ACE, exerts various effects based on the receptor type it binds to, with oxidative stress, inflammation, fibrosis, and vasoconstriction being effects associated with the Angiotensin I receptor (AT1R), and when Ang II binds to the Angiotensin II receptor (AT2R), the opposite effects are evoked [7,16,17,18]. ACE2, discovered in 2000, counterbalances Ang II activity by converting it to Ang (1–7), promoting vasodilation, antioxidant, and antiproliferative effects [19,20,21].

Rodents infected with SARS-CoV-1 previously resulted in lung injury mediated by ACE2 downregulation and impaired degradation of angiotensin II [22]. Also, a deletion of ACE2 led to more severe influenza disease courses [23]. Moreover, significantly greater numbers of ACE2-positive cells were counted in the lungs from patients who died of COVID-19 or influenza compared with uninfected control subjects [24].

Concerns were raised about the use of RAAS inhibitors (RAASi), such as ACE inhibitors (ACEi) and angiotensin receptor blockers (ARBs), in COVID-19 patients, due to the potential upregulation of ACE2. RAASi in the context of antihypertensive therapy could play a negative key role in the mechanism of SARS-CoV-2 infection by theoretically leading to a higher number of binding sites for SARS-CoV-2 at the cellular membrane, mediating an infection with the virus, or even aggravating the course of the disease [25,26,27]. However, studies like the BRACE CORONA trial indicated no need to discontinue RAASi in COVID-19 patients, with some research even suggesting potential benefits [3,28,29,30].

No major changes in RAAS-activity in the plasma of COVID-19 patients per se were found so far, but in COVID-19 patients treated with RAASi on the other hand, an increased plasma ACE2 activity could be observed, which was not observed in non-COVID-19 patients treated with RAASi [31,32,33]. The clinical significance of upregulated plasma ACE2 during RAASi therapy remains unclear [33,34,35]. However, because RAASi—especially angiotensin-converting-enzyme inhibitors (ACEi) and angiotensin-receptor blockers (ARB)—have been shown to improve endothelial dysfunction, to reduce large artery wall thickening, and to regress smooth muscle cell hypertrophy in the past [25], a potential benefit of RAASi therapy in COVID-19 patients could not be ruled out so far. Therefore, it is of great interest to identify further knowledge on this topic.

Of note, the distinction between tissue ACE2 (cACE2) and plasma ACE2 (=soluble ACE2, sACE2) is crucial, as sACE2 retains an intact SARS-CoV-2 binding site after being shed from cACE2, leading to various theories about its role in the virus’s mechanism of infection and potential treatment strategies [27,31,32,36,37,38].

Thus, the role of sACE2 in severe COVID-19 is unclear. On the one hand, it facilitates virus entry into cells [37], on the other hand, sACE2 (as opposed to membrane-bound ACE2) is hypothesised to neutralize the virus when binding to it [39,40,41] or inhibits cell entry [42].

It might be possible that RAASi-induced upregulation of plasma ACE2 has a favourable effect on COVID-19 patients by improving arterial stiffness, lowering pulse wave velocity (PWV), and, therefore, lowering the risk for major adverse cardiac events.

We, therefore, aimed to assess the relation of RAASi, sACE2, and vascular function in acutely ill patients with COVID-19 in comparison with acutely ill patients without COVID-19.

## 2. Materials and Methods

### 2.1. Study Design and Objectives

This prospective single-centre observational study aimed to examine and compare the changes in sACE2 levels in COVID-19 and non-COVID-19 patients treated at an Emergency Department. Additionally, the study investigated the effect of chronic intake of renin−angiotensin−aldosterone system inhibitors (RAASi) on sACE2 levels in these patient groups and explored the potential positive effects of sACE2 upregulation on arterial stiffness, measured via PWV.

The primary research questions were as follows:

1. What effect does COVID-19 have on sACE2 levels?
2. What effect does RAASi therapy have on sACE2 levels in COVID-19 patients compared to non-COVID-19 patients?
3. What effect does upregulated sACE2 have on arterial stiffness, measured via PWV, in acutely ill COVID-19 patients?

### 2.2. Study Population and Data Acquisition

The study was conducted at the Department of Emergency Medicine, Medical University of Vienna, Austria—a tertiary care academic medical centre. Between November 2020 and May 2021, adult emergency department patients who had a SARS-CoV-2 infection confirmed via reverse-transcription polymerase chain reaction (COVID-19 group) on the one hand, and patients initially suspected of COVID-19 but then tested negative (non-COVID-19 group) on the other hand were included. Eligible patients received PWV and sACE2 measurements during their emergency department stay. Ankle-brachial index (ABI) and PWV measurements were conducted (BOSO ABI Systems 100 PWV^®^, Bosch and Sohn GmbH, Jungingen, Germany), and yielded two ABI values (left and right) and two brachial-ankle PWV (baPWV) values (left and right). For the analysis, the more pathological values were used (lower ABI value, higher PWV value). In cases where measurements were only successful on one side, that value was used. Carotid-femoral PWV (cfPWV) values were calculated from the baPWV values by the measuring device. sACE2 (in nanograms per millilitre [ng/mL] was measured from the blood samples routinely during the patients’ emergency department stay via the Human ACE-2 Duo ELISA set (R&D Systems, Minneapolis, MN, USA). The number of non-COVID-19 patients in who sACE2 was measured does not equal the one of the COVID-19 patients because the funding for additional measurement kits was not available anymore. Demographic and clinical data were collected via direct patient contact and from the electronic patient information system. Ethical approval was obtained from the Ethics Committee of the Medical University of Vienna (evaluation numbers 2197/2017 and 1394/2022), and all participants signed a written informed consent form.

### 2.3. Statistical Analysis

Descriptive characteristics are expressed using counts, percentages, means, and standard deviations (SDs). Categorical data are expressed with absolute and relative frequencies. For metric variables, medians with interquartile ranges (IQRs) are given. To verify the assumption of a normal distribution of the parameters, Q−Q plots were generated. For a comparison of groups, Chi-square or Fisher exact tests were used. The Wilcoxon rank-sum test was used to analyse the differences in numerical data. All tests were two-sided and *p*-values of <0.05 were considered statistically significant. For analyses, Microsoft Excel Version 16.9 (22031300) and the software R (RStudio Version 1.1456, RStudio Inc., Boston, MA, USA) were used.

## 3. Results

We included 152 patients (76 or 50% female; 62 [50–77] years, BMI 27.1 [24.6–31.7]), with 103 (68%) diagnosed with COVID-19, and the most common comorbidity being arterial hypertension. No significant differences in basic demographics, comorbidities, or chronic RAASi intake were noted between COVID-19 and non-COVID-19 patients (Supplementary Table S1). None of the included patients were critically ill. Clinically, the non-COVID-19 group showed respiratory infection from other pathogens that were not measured (thus, not SARS-CoV-2, respiratory syncytial virus, or influenza). Apart from ABI, the measured vascular function parameters also showed no differences (see Table 1). Median sACE2 levels were tendentiously, but not significantly, higher in COVID-19 patients (0.485 [0.364–1.329)], compared with non-COVID-19 patients (0.458 [0.356–1.138]; *p* = 0.70).

**Figure 1:**
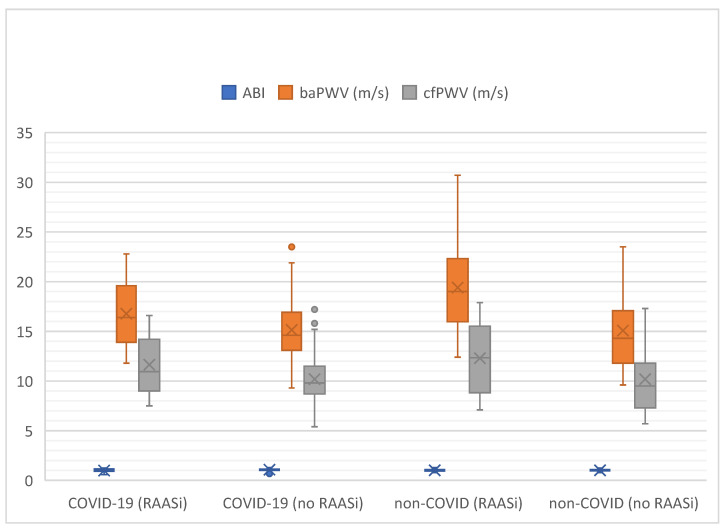
Overview of ABI and PWV values in all cohorts. ABI = ankle-brachial index; PWV = pulse-wave velocity; baPWV = brachial-ankle pulse-wave velocity; m/s = meters per second; cfPWV = carotid-femoral pulse-wave velocity 2; RAASi = renin−angiotensin−aldosterone system inhibitors.

The comparisons of vascular function parameters in the groups COVID-19 with and without chronic RAASi and non-COVID-19 with and without chronic RAASi are listed in Table 2 and depicted in Figure 1. Regarding sACE2 values, no significant differences were noted (Table 2). Assessing baPWV values in regard to the mentioned study subgroups, we saw a significant difference between the four groups (*p* = 0.015); interestingly, this difference was not reproducible for cfPWV (*p* = 0.133; see Supplementary Figure S1). A probability density function of baPWV distribution in patients with and without COVID-19 and with and without RAASi intake showed that the most patients in the lowest baPWV (least pathological) range were in the non-COVID-19 group without RAASi inhibitors, whereas the group with the most patients in the highest baPWV range (most pathological) were in the COVID-19 group with RAASi intake.

**Figure 2:**
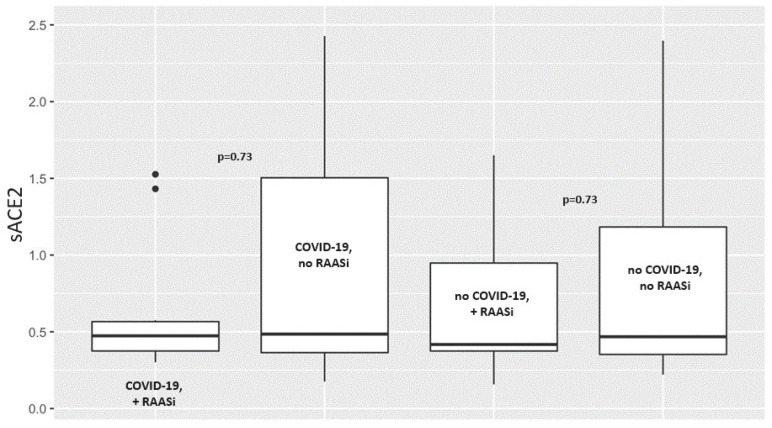
sACE2 values of COVID-19 and non-COVID-19 patients with and without RAASi. sACE2 = soluble angiotenin-converting enzyme 2; RAASi = renin−angiotensin−aldosterone system inhibitors.

Median sACE2 levels did not significantly differ between COVID-19 patients with RAASi therapy (0.474 [0.375–0.566]) and those without RAASi therapy (0.485 [0.364–1.504], *p* = 0.73). The same was found for the non-COVID-19 groups (see Table 2 and Figure 2).

## 4. Discussion

### 4.1. sACE2 Levels in COVID-19

The study aimed to evaluate changes in sACE2 levels in COVID-19 patients and non-COVID-19 patients, particularly in the context of RAASi therapy. The sACE2 levels were slightly higher in the COVID-19 group when compared with the non-COVID-19 group, but not to a statistical significance. This observation aligns with the potential role of sACE2 in responding to increased inflammation caused by Ang II—AT1R binding, as indicated by previous research [7,17,18,34]. However, our findings do not correlate with the observations of Kintscher et al. [33], who observed no major changes in RAAS-activity in the plasma of COVID-19 patients per se, which also included sACE2 activity. Kornilov et al. [43], on the other hand, found that patients with cardiometabolic disease had higher sACE2 levels and proposed chronic inflammation to play a major role in sACE2 elevation, which points to a possible shared pathway in patients with cardiometabolic disease and COVID-19.

### 4.2. RAASi and sACE2 in COVID-19

We also investigated the effect of RAASi therapy on sACE2 levels. It was hypothesised that sACE2 might be upregulated due to RAASi intake, which could have a favourable effect on COVID-19 patients by improving the arterial stiffness, lowering the pulse-wave velocity, and, therefore, lowering the risk for major adverse cardiac events. However, in our data, RAASi therapy did not lead to an upregulation of sACE2, but rather the opposite: sACE2 was slightly upregulated in patients who did not have RAASi as their antihypertensive therapy. We suggest that an upregulation in sACE2 does take place in COVID-19 patients when compared with non-COVID-19 patients, which does not—other than hypothesised by other studies [31,32,33]—seem to be RAASi-induced, but rather to be caused by the oxidative stress and inflammation that comes with the disease itself [9,13,14].

### 4.3. Arterial Stiffness

Arterial stiffness has been discussed to increase as a consequence of indirect endothelial damage induced by SARS-CoV-2 through the release of hyperinflammation and cytokine release, which reduces NO availability by the cytokine cascade [9,13,14]. PWV has previously been reported to be higher in COVID-19 patients than in non-COVID-19 individuals [44]. While no significant difference was found in the data at hand, our results suggest that COVID-19 patients exhibited more pathological values in all categories (ABI, baPWV, and cfPWV) when compared with non-COVID-19 patients. This indicates a potential increase in arterial stiffening in COVID-19 patients.

We also observed that RAASi therapy did not lead to improved arterial stiffness in COVID-19 patients; in fact, patients with RAASi therapy showed worse arterial stiffness, which may, however, be attributed to the underlying chronic multimorbidity of patients treated with RAASi.

Upregulated sACE2 did not have a beneficial effect on arterial stiffness in COVID-19 patients either; patients with higher sACE2 levels exhibited worse PWV, potentially due to sACE2 levels being more upregulated in response to the oxidative stress and inflammation that comes with COVID-19 [9,13,14].

### 4.4. Research Outlook

Further research is required to understand the mechanism of action of sACE2 in more detail. To reduce selection bias, a matched sampling of the study cohort by common comorbidities can be performed. Additionally, a direct measurement of Ang (1–7) and Ang (1–9) levels may provide more conclusive evidence of the potential beneficial effects of upregulated sACE2.

### 4.5. Relevance for Medical Practice

The findings of this study provide insights into the use of RAASi therapy in COVID-19 patients. The study does not support the claim to discontinue RAASi therapy in COVID-19 patients, as it plays a crucial role in protecting cells from chronic inflammation and apoptosis.

### 4.6. Limitations

A cohort of 103 COVID-19 patients were included in the data analysis, whereas only 49 non-COVID-19 patients were eligible for inclusion due to funding reasons (see Methods). Furthermore, the groups of patients with RAASi intake and without RAASi intake in both cohorts differed in numbers. Moreover, our data analysis was performed as a single-centre observational study. However, our tertiary care centre has a wide catchment area, possibly balancing out bias. Due to missing data, not all parameters could be evaluated in every patient. RAASi are commonly used in patients with systemic hypertension, heart failure, or kidney disease. PWV values in patients with RAASi intake might thus have been influenced by common comorbidities in this population, and a comparison of RAASi and non-RAASi groups could thus have been biased. Also, no matching by age or sex was possible, leading to sex-related differences in regard to cardiovascular diseases potentially further impacting on results [45]. Last, RAASi do not only include ACEi and ARBs, but also Mineralocorticoid Receptor Antagonists (MRA) and Direct Renin Inhibitors (DRI) [46]. Due to a different mechanism of action, MRA and DRI were not included in the data analysis. Nevertheless, the intake of MRA or DRI might have had an influence on PWV.

## 5. Conclusions

This prospective observational study found sACE2 levels to be upregulated in COVID-19 patients, most likely due to oxidative stress and inflammation caused by the disease. However, RAASi therapy did not account for further sACE2 upregulation. Rather, the absence of RAASi therapy did so, possibly due to a lack of additional protection from inflammation that RAASi intake can provide through inhibiting AT1R binding of Ang II. Upregulated sACE2 seemed to have beneficial effects on arterial stiffness, seen by lower PWV values in both COVID-19 and non-COVID-19 patients. Noted elevated PWV values in patients with RAASi therapy were likely caused by the underlying multimorbidity. Thus, this study supports the suggestion to sustain RAASi therapy in COVID-19 patients as it can play a role in protecting cells from chronic inflammation and apoptosis.

## Figures and Tables

**Table 1:** ABI, PWV, and sACE2 values in COVID-19 patients versus non-COVID-19 patients. ABI = ankle-brachial index; N = number of total measurements/population; IQR = interquartile range; x = number of patients with certain value; baPWV = brachial-ankle pulse-wave velocity, m/s = meters/second; cfPWV = carotid-femoral pulse-wave velocity; sACE2 = soluble angiotensin-converting enzyme 2; ng/mL = nanograms per millilitre.

	COVID-19 (N = 103)	Non-COVID-19 (N = 49)	*p*-Value
ABI [IQR]	1.08 [0.99–1.14]	1.0 [0.96–1.10]	0.03
baPWV, m/s; [IQR]	14.9 [13.2–18.6]	15.1 [12.4–19.5]	0.83
baPWV ≥ 16, m/s; x of N (%)	31 of 87 (36%)	22 of 48 (46%)	0.33
cfPWV, m/s; [IQR]	10.1 [8.7–12.6]	9.6 [7.8–13.1]	0.77
cfPWV ≥ 10, m/s; x of N (%)	44 of 83 (53%)	21 of 43 (49%)	0.80
sACE2, ng/mL; [IQR]	0.485 [0.364–1.329]	0.458 [0.356–1.138]	0.70

**Table 2:** ABI, PWV, and sACE2 levels in COVID-19 patients with RAASi vs. COVID-19 patients without RAASi. ABI = ankle-brachial index; PWV = pulse-wave velocity; sACE2 = soluble angiotenin-converting enzyme 2; RAASi = renin−angiotensin−aldosterone system inhibitors; N = number of total measurements/population; baPWV = brachial-ankle pulse-wave velocity; cfPWV = carotid-femoral pulse-wave velocity; ng/mL = nanograms per millilitre.

	COVID-19 with RAASi (N = 32)	COVID-19 Without RAASi (N = 71)	*p*-Value	Non-COVID-19 with RAASi (N = 12)	Non-COVID-19 Without RAASi (N = 37)	*p*-Value
ABI [IQR]	1.06 [0.91–1.15]	1.09 [1.01–1.13]	0.13	1.01 [0.94–1.10]	1.00 [0.96–1.10]	0.95
baPWV, m/s [IQR]	16.4 [13.9–19.6]	14.6 [13.1–16.9]	0.06	19.0 [16.0–22.3]	14.3 [11.8–17.1]	0.01
baPWV ≥ 16, m/s x of N (%)	12 von 23 (52%)	19 of 64 (30%)	0.09	75% (9 of 12)	36% (13 of 36)	0.044
cfPWV, m/s [IQR]	11.0 [9.0–14.2]	10.2 [8.7–11.5]	0.06	12.4 [8.8–15.5]	9.5 [7.3–11.8]	0.15
cfPWV ≥ 10, m/s x of N (%)	15 von 22 (68%)	29 of 61 (48%)	0.16	60% (6 of 10)	45% (15 of 33)	0.66
sACE2, ng/mL [IQR]	0.474 [0.375–0.566]	0.485 [0.364–1.504]	0.73	0.417 [0.375–0.948]	0.468 [0.353–1.183]	0.73

## Data Availability

Data can be obtained from the corresponding author upon reasonable request. This is in accordance with national law and organisational regulations.

## Supplementary Materials

The following supporting information can be downloaded at: https://www.mdpi.com/article/10.3390/jcm14072233/s1: demographics. Figure S1: BaPWV and cfPWV values of COVID-19 and non-COVID-19 patients Supplementary Table S1: Basic with and without RAASi.
