# Supplementary material for: The Relation of Angiotensin-Converting Enzyme 2, Renin-Angiotensin-Aldosterone System Inhibitors, and Arterial Stiffness in Acute COVID-19 Emergency Department Patients—A Prospective Observational Study
Source: J Clin Med. 2025 Mar 25;14(7):2233. doi: 10.3390/jcm14072233 (PMC11989675; doi:10.3390/jcm14072233)
Supplement: Supplementary file 1 [file jcm-14-02233-s001.zip › jcm-3499773-supplementary.pdf]

	Total study population (n=152)	COVID-19 (n=103)	Non-COVID-19 (n=49)	p-value
Gender (male), n (%)	76	49 (48)	27 (55)	0.208
Age (years), median (IQR)	62 (50-77)	61 (50-76)	63 (52-80)	0.592
BMI (kg/m2), median (IQR)	27.1 (24.6-31.7)	26.3 (24.6-31.7)	27.2 (25.0-31.9)	0.183
AHTN, n (%)	62 (41)	41 (40)	21 (42)	0.388
HLP, n (%)	35 (23)	23 (22)	12 (24)	0.471
DM II, n (%)	38 (25)	24 (23)	14 (29)	0.199
CKD, n (%)	12 (8)	8 (8)	4 (8)	0.812
PAD, n (%)	12 (8)	7 (7)	5 (10)	0.301
AF, n (%)	14 (9)	9 (9)	5 (10)	0.633
CAD, n (%)	29 (19)	19 (18)	10 (20)	0.099
Thyroid disease, n (%)	23 (15)	15 (15)	8 (16)	0.461
History of stroke, n (%)	11 (7)	8 (8)	3 (6)	0.375
History of myocardial infarction, n (%)	17 (11)	10 (10)	7 (14)	0.103
Past or active smoker, n (%)	44 (29)	31 (30)	13 (27)	0.171
RAASi, n (%)	85 (56)	32 (31)	12 (25)	0.403
ACEi, n (%)	30 (20)	12 (12)	4 (8)	0.512
ARB, n (%)	53 (35)	20 (19)	8 (16)	0.082

Supplementary Table S1: Basic demographics. COVID-19 = coronavirus disease 2019. BMI = body mass index; AHTN = arterial hypertension; HLP = hyperlipidemia; DM II = diabetes mellitus type 2; CKD = chronic kidney disease; PAD = peripheral artery disease; AF = atrial fibrillation; CAD = coronary artery disease; RAASi = renin-angiotensin-aldosterone system inhibitors; ACEi = angiotensin converting enzyme inhibitors; ARB = angiotensin receptor blockers.

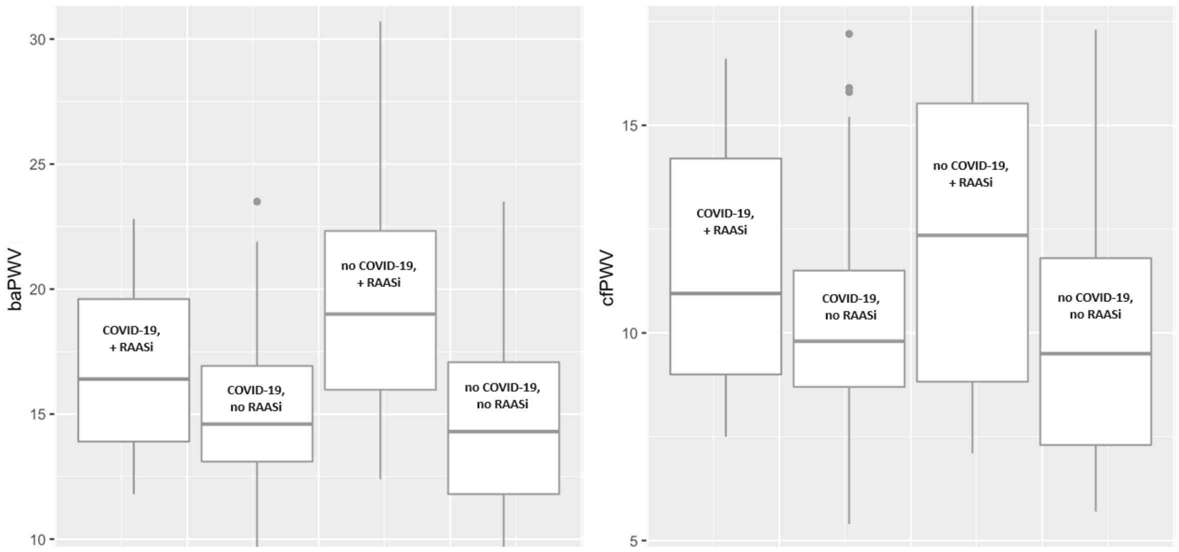

Supplementary Figure S1: BaPWV and cfPWV values of COVID-19 and non-COVID-19 patients with and without RAASi. BaPWV=brachial-ankle pulse-wave velocity; cfPWV = carotid-femoral PWV; RAAS=renin-angiotensin-aldosterone system.
